# Evaluating Treatment and Safety Outcomes of a Shorter Regimen for Drug-Resistant TB in Nigeria: An Implementation Research Study

**DOI:** 10.3390/tropicalmed11030084

**Published:** 2026-03-21

**Authors:** Victor Babawale, Clement Adesigbin, Corinne S. Merle, Vanessa Veronese, Fatimata Bintou Sall, Benjamin Seydou Sombie, Eunice Nnaisa Jiya-Chitumu, Chizaram Onyeaghala, Adegboyega Moses Oyefabi, Rotimi Samuel Owolabi, Osman Eltaye, Olusoji Ige, Ogiri Sam, Obioma Akaniro, Adebola Lawanson, Victor Ombeka, Muse Fadeyi

**Affiliations:** 1 National Tuberculosis and Leprosy Control Programme, Federal Ministry of Health, Abuja 900288, Nigeria; 2 The Special Programme for Research and Training in Tropical Diseases (TDR), World Health Organisation, 1211 Geneva, Switzerland; 3 National Tuberculosis and Leprosy Training Center Saye-Zaria, Zaria 810241, Nigeria; 4 Department of Internal Medicine, University of Port Harcourt Teaching Hospital, Port Harcourt 500102, Nigeria; 5 Department of Community Medicine, Kaduna State University, Kaduna 800283, Nigeria; 6 University of Abuja Teaching Hospital, Gwagwalada, Abuja 902101, Nigeria; 7 Damien Foundation Belgium, Ibadan 200273, Nigeria; 8 University College Hospital, Ibadan 200285, Nigeria; 9 World Health Organization, Abuja 900211, Nigeria

**Keywords:** multi-drug-resistant tuberculosis, TB treatment effectiveness, shorter, all oral TB regimens, Nigeria

## Abstract

The introduction of significantly shorter, all-oral regimens has significantly shifted the management of drug-resistant tuberculosis (DR-TB) towards a more tolerable and patient-centred therapeutic approach that aims to enhance treatment adherence, clinical outcomes, and quality of life among patients. Nigeria has gradually adopted this all-oral, shorter regimen, but the impact of this regimen in programmatic settings has not yet been studied. In 2022, a longitudinal, two-armed cohort study was conducted to explore the effectiveness, safety, and feasibility of the all-oral shorter regimen in the programmatic management of RR/MDR-TB in Nigeria. Consenting and eligible RR/MDR-TB patients receiving the all-oral regimen (intervention group) in four states were consecutively enrolled and compared to those receiving the standard of care (SOC). Treatment effectiveness, proportion, and 95% confidence intervals of favourable and unfavourable outcomes were measured at the end of treatment and during follow-up (six and 12 months post-treatment). In total 383 Participants were followed monthly throughout the 9–12-month treatment phase and then reassessed at 6 and 12 months after treatment completion, giving a total possible observation period of up to 24 months (185 received the intervention and 198 the standard of care). At the end of follow-up, there was a higher but non-significant proportion of favourable outcomes among the intervention vs. SOC group (80% vs. 69.7%); a higher proportion of favourable outcomes was also noted at the end of treatment among intervention participants (81.1 vs. 76.8%). Around one third of patients reported at least one serious adverse event (SAE), with no significant differences between arms, and none were deemed related to the use of medication. Intervention participants reported greater improvements in health-related quality of life between baseline and four months compared to those receiving the SOC. These findings support the programmatic use of all-oral shorter treatment for RR/MDR-TB as a regimen that is effective, tolerable, safe, and associated with enhanced health-related quality of life for patients in Nigeria.

## 1. Introduction

The emergence of drug-resistant TB is a significant public health problem and an obstacle to achieving effective global TB control [1,2]. An estimated 400,000 people developed rifampicin-resistant or multidrug-resistant TB (RR/MDR-TB) in 2023, leading to an estimated 150,000 deaths [3,4,5,6]. Nigeria is among the 30 highest burden countries for MDR/RR-TB, and while the estimated number of incident RR/MDR-TB cases have almost halved over the past decade, challenges such as sub-optimal practice of and adherence to directly observed treatment (DOT), poor infection prevention and control measures, and increasing community transmission of drug-resistant TB contribute to ongoing transmission in Nigeria [7,8,9,10]. In line with the World Health Organization (WHO) guidance, Nigeria has recently scaled up the expansion of the rapid molecular diagnostic platforms to enhance diagnostic accuracy and timely TB diagnosis and the detection of drug resistance.

In 2019, the WHO released new consolidated guidelines on the treatment of DR-TB, which recommended the transition to an all-oral, shorter regimen for patients with RR/MDR-TB, without resistance to fluoroquinolones [11]. The use of a shorter, all-oral regimen containing linezolid for those resistant to fluoroquinolones (BPaL regimen) was also advised under operational research conditions [12,13,14,15,16,17]. Consequently, these new regimens were adopted by the National Tuberculosis and Leprosy Control Programme (NTBLCP) of Nigeria. This implementation research study was conducted to evaluate the effectiveness, safety, and impact on the quality of life of the all-oral shorter regimens delivered under programmatic conditions in Nigeria.

## 2. Materials and Methods

### 2.1. Study Design

A prospective cohort study with two parallel arms was undertaken among MDR/RR-TB patients receiving the all-oral shorter MDR/RR-TB regimen compared to a comparison group receiving the standard of care (SOC). The study was conducted in eight selected states across Nigeria, with four randomly selected as the intervention group for the all-oral shorter regimen (Oyo, Plateau, Ebonyi, and Rivers) and the other four states as the control group for SOC (Ogun, Nasarawa, Abia, and Kaduna). The ShORRT research package, developed by The Special Programme for Research and Training in Tropical Diseases (TDR), was adapted to the Nigerian context for the development of the research protocol and data collection tools [18].

### 2.2. Participants

The study population comprised patients evaluated at selected TB clinics with bacteriologically confirmed rifampicin-resistant tuberculosis (RR-TB) or those clinically suspected of MDR/RR-TB, including children with a history of close contact with a confirmed MDR/RR-TB case. Children likely to be MDR/RR-TB based on a history of close contact with a confirmed MDR/RR-TB case were also considered. Eligibility criteria were defined as follows: willing and able to give informed consent (including signed or witnessed consent for illiterate patients and signed or witnessed consent from a parent or legal guardian for minors), and evidence of bacteriologically confirmed TB and resistance to at least rifampicin. Patients whose DST results identified resistance to fluoroquinolones, who were unable to take oral medication, had allergies or were taking other medications that contraindicated any of the medicines under investigation, and/or patients who had a corrected QT (QTc) interval of >500 msec were considered ineligible.

### 2.3. Treatment Regimens

Patients received either the modified all-oral shorter regimen (intervention) or the WHO-approved RR/MDR-TB regimen in use in Nigeria at the time (SOC), according to the regimen implemented in their state. The all-oral shorter regimen consisted of a 4–6-month intensive phase with bedaquiline (Bdq), high-dose levofloxacin (hLfx), linezolid (Lzd), and clofazimine (Cfz), followed by a 5-month continuation phase with Bdq (for the first two months), hLfx, Lzd, and Cfz.

The standard of care regimen consisted of 4–6 months of Bdq, moxifloxacin (Mfx), clofazimine (Cfz), high-dose isoniazid (Hh), prothionamide (Pto), ethambutol (E), and pyrazinamide (Z), followed by 5 months of Mfx, Cfz, E, and Z. Dosing was weight-based (Table 1).

Both regimens were fully oral and had a total duration of approximately 9–12 months. The main differences between regimens were the fluoroquinolone backbone (hLfx vs. Mfx) and the inclusion of linezolid with clofazimine in the all-oral shorter regimen versus high-dose isoniazid and prothionamide in the SOC regimen, reflecting efforts to improve tolerability and simplify treatment while maintaining efficacy.

### 2.4. Study Procedures

Patients presenting to any one of the eight study sites during the recruitment period and found to have evidence of resistance to rifampicin by either culture or molecular DST (i.e., Xpert MTB/RIF or Line Probe Assay) were assessed for enrollment and asked to provide informed consent. Consenting and eligible patients underwent the following baseline screening: collection of patient demographics and medical history; clinical evaluation, bacteriological and other laboratory testing; a chest X-ray and a 12-lead electrocardiogram (ECG) (or single-channel portable monitors). Chest X-ray (CXR) was used to determine the extent of pulmonary disease based on the proportion of lung fields involved. For ease of interpretation, we categorized radiographic extent into three ordered groups: category A (<25% of lung fields affected), category B (25–49% of lung fields affected), and category C (≥50% of lung fields affected or bilateral/multilobar disease). Higher categories therefore indicate more extensive lung damage.

After screening, treatment was initiated and patients were followed up monthly during the 9–12-month treatment phase. Monthly follow-up comprised clinical examination, collection of sputa for smear and culture, laboratory testing (hematology and serum liver enzymes, thyroid-stimulating hormone (TSH) at months three and six only, and ECG. DST was performed at baseline and after any reversion identified during treatment, and/or if any culture was found positive during the post-treatment follow-up or after conversion. At each visit, patients were asked about any adverse events experienced since the previous visit for monitoring and management.

At the end of the treatment phase, patients were followed for an additional 12 months and asked to return at six and 12 months post-treatment completion (or sooner in the event of TB clinical symptoms) for clinical assessment and sputum collection for smear and culture; culture-positive patients received follow-up DST examination (either culture or molecular), LPA, and CXR which was also performed among patients under suspicion of recurrent TB.

Disability assessment was conducted at baseline, end of treatment, and during follow-up using the Modified Medical Research Council Dyspnea Scale (mMRC), which is used to assess the degree of baseline functional disability due to dyspnoea by assigning a numerical score (0–4) to various statements reflecting the degree of breathlessness [19]. Health-related quality of life was also assessed at baseline, month four, end of treatment, and at 12-month follow-up using the EQ-5D-5L tool (Table 2).

### 2.5. Study Outcomes

The primary outcome was the effectiveness and safety of the intervention regimen compared to SOC. Effectiveness was defined as those with a favourable treatment outcome (patients who have completed treatment with no evidence of failure but who do not have bacteriological evidence of cure) or cure (patients who have completed treatment, have no evidence of failure, and at least two consecutive negative cultures at least 30 days apart at the end of treatment), and without recurrence at 12 months after the end of treatment. The primary safety outcome was defined as the proportion of patients reporting any occurrence of a serious adverse event (SAE) during treatment and up to six months after the end of the treatment.

Secondary outcomes assessed were as follows: treatment adherence (defined as patients missing less than 10% of treatment doses during the course of their therapy); proportion of patients self-reporting experiences of disability due to breathlessness using the mMRC Dyspnoea scale to measure the degree of disability that breathlessness poses on day-to-day activities on a scale from 0 to 4; [19]; proportion cured without permanent disability: a combined outcome, using the mMRC scale based on which patients with a score above 2 are considered permanently disabled in terms of their pneumological function, in addition to all serious adverse events by system organ class that are not resolved at the end of treatment, and; health-related quality of life: measured at baseline, month 4, end of treatment, and follow-up using the EuroQol visual analogue scale (EQ-VAS), a standardized tool that records patients’ self-rated health on a vertical scale from 0 (“the worst health you can imagine”) to 100 (“the best health you can imagine”). This tool was used to assess perceptions of health and functioning across five dimensions at baseline, month four, end of treatment, and at follow-up [19].

### 2.6. Recruitment and Sample Size

The target sample size of 400 participants (200 per arm) was determined based on the existing and projected case load and potential recruitment rate of bacteriologically confirmed DR-TB patients across each of the selected study sites. At each site, all consecutively presenting patients with bacteriologically confirmed RR/MDR-TB who met the eligibility criteria during the recruitment period were invited to participate until the overall sample size was reached. This approach reflects routine programmatic enrolment rather than random sampling of individuals.

### 2.7. Data Collection and Analysis

Socio-demographic, clinical, and laboratory data were collected from all participants using routine forms and registers and study-specific forms. Clinical data, including adverse events, laboratory data such as results of DST and other microbiological test results, were recorded onto the patient’s medical records and/or treatment cards and acted as the source documents. Individual patient case report forms (CRFs) were developed for the study and patient data was abstracted from source documents onto CRFs on at least a monthly basis by research assistants working at the study sites.

An electronic database, REDCap, was created and used to support data storage and management. Data was transposed from the CRFs to the REDCap database by a focal point at each of the study sites. Research staff at the national level had access to the database and were responsible for regular data review, management, and the identification of discrepancies. Any issues related to data quality or completeness were addressed directly with the relevant study team.

Descriptive statistics were used to summarize patient characteristics at baseline, and significance tests (Chi-square test, Fisher’s exact test, Mann–Whitney test, or Kruskal–Wallis tests as appropriate) were used to identify significant differences between the two treatment groups, including the occurrence of AEs of interest during the treatment phase. For treatment effectiveness, the proportion and 95% confidence intervals (CIs) of favourable and unfavourable treatment outcomes were measured at the end of treatment and during follow-up. Risk ratios were calculated to evaluate the differences in outcomes across the two groups and were adjusted by selected patient and clinical variables to account for possible confounding. Stata version 17 (StataCorp LLC, College Station, TX, USA) was used to perform all statistical analyses.

### 2.8. Ethics

This study received ethical approval from the WHO Research Ethics Review Committee (Protocol ID: ERC.0003305) and the National Health Research Ethics Committee (NHREC/01/01/2007 -01/06/2020). Written (or witnessed in the case of illiterate or minor participants) informed consent was obtained from all patients. All participant information was handled in accordance with applicable ethical and data protection standards. Personal identifiers were removed from study datasets and replaced with unique study identification numbers to ensure anonymity. Data were stored in password-protected electronic REDCap databases accessible only to authorized study personnel. Hard-copy CRF documents were kept in locked cabinets within secure offices. No identifiable patient information was included in the analysis or reporting.

## 3. Results

**Figure 1:**
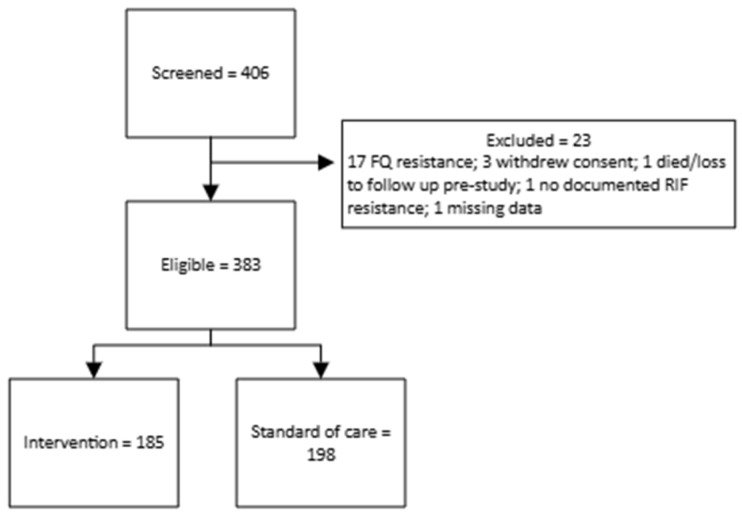
Flowchart of participant recruitment and enrollment.

Between the 3rd of November 2020 and the 25th of May 2022, 406 patients were screened, of whom 17 were excluded on the basis of identified FQ resistance, three withdrew their consent, one died before treatment commenced, one did not have evidence of rifampicin resistance, and one had no data. Of the 383 remaining patients included in the analysis, 185 patients were enrolled at the intervention sites and received the all-oral shorter regimen and 198 patients were enrolled at the control sites and received the standard of care regimen (Figure 1).

Overall, patients had a median age of 35 (IQR: 25–45), were male (64.0%), new TB cases (77.8%), were previously treated with first-line drugs (92.9%), had a median BMI of 18.4, reported no existing neuropath (98.4%), returned a negative pregnancy test for female patients (87.6%), tested negative for HIV (90.1%), Hepatitis B and C (94.3 and 96.9%, respectively), and diabetes (94.3%). Measurements for the assessed clinical variables are presented in Table 3. The disability assessment identified that the majority of patients reported breathlessness with strenuous exercise (64.8%) and one-fifth reported breathlessness with moderate exertion, such as walking quickly or on an incline (23.9%). Patients in the two treatment groups differed significantly across the following variables: age group, patient type, Hepatitis B status, glucose, and creatinine values (Table 3).

The majority of patients were sputum smear-negative (48.6%) and culture-positive (59.5%). All patients were resistant to rifampicin, almost all had documented sensitivity to fluoroquinolone (84.9%), and roughly half were sensitive to isoniazid (48.0%). Just under half had evidence of cavitation on chest X-ray (45.7%) and two-fifths were classified as disease severity category B (41.5%). Patients in the two treatment groups differed significantly across the following clinical variables: sputum smear status, fluoroquinolone and isoniazid sensitivity status, cavitation on CXR, and extent of disease category (Table 4).

### 3.1. Effectiveness of Shorter All-Oral Regimen

At the end of the follow-up period, the intervention group had a higher but non-significant proportion of favourable treatment outcomes compared to the standard regimen (80.5% vs. 69.7%; *p* > 0.05). One episode of recurrent TB was noted in each group. Similar proportions in both arms were considered cured without permanent disability (Table 5).

Patients in the intervention group also had a higher proportion of favourable outcomes at the end of treatment: 81 vs. 76.8% among patients receiving the SOC. Five patients in the intervention group had treatment failure compared to three in the SOC regimen group (2.7 vs. 1.5%, respectively). Of the five failures in the intervention group, four were due to bacteriological reversion of sputum culture after conversion to negative, and one was due to a permanent change of at least two anti-TB drugs in the regimen because of adverse drug reactions (ADRs). All three failures in the SOC regimen were due to a lack of culture conversion after 4 months of treatment. Overall treatment adherence was high across both arms (Table 6).

### 3.2. Safety Outcomes

During the treatment phase, 344 patients overall (90.0%), with similar proportions across the two groups (87 vs. 92% intervention vs. SOC), experienced AEs. Overall, 1277 individual AEs were reported or an average of four per patient (IQR 2–5) and two per patient for AEs of interest among patients experiencing at least one AE during the treatment phase (IQR 2–3; Table 7).

The most frequently reported events were elevated liver enzymes (37.7%), QTc prolongation (27.6%), and anemia (21.1%), followed by thrombocytopenia (11.4%). Other events were uncommon, each accounting for less than 2% of reported events. Regarding severity, most AEs were grade 1 (45.9%) or grade 2 (38.2%), while grade 3 and grade 4 events represented 12.4% and 3.5% of events, respectively. The distribution of AE types and severity grades was broadly similar between treatment groups (Supplementary Data, see Appendix A Table A1).

When considering the proportion of participants experiencing at least one AE of interest, 279 participants (73.4%) experienced elevated liver enzymes, 211 (55.5%) experienced QTC prolongation, and 184 (48.4%) experienced anemia. The proportion of participants with elevated liver enzymes was significantly higher in the standard regimen group compared with the all-oral shorter regimen group (79.4% vs. 66.9%, *p* = 0.008). Conversely, peripheral neuropathy occurred only in the all-oral shorter regimen group (3.9% vs. 0.0%, *p* = 0.005). No statistically significant differences were observed between treatment groups for the other adverse events, including anemia, thrombocytopenia, leukopenia, QTc prolongation, anxiety, depression, optic neuritis, and ototoxicity (Supplementary Data, see Appendix A Table A2).

#### Serious Adverse Events

In total, there were 32 patients who experienced at least one SAE with a non-significant difference in the proportion of SAE patients between the two groups. The majority of SAEs were deaths, which occurred among 11 patients in the all-oral group and 17 in SOC (85.7% and 81.0%, respectively). None of the SAEs were determined as related to the medication, 69.7% were deemed unrelated, and 30.3% were unable to be attributed due to insufficient data (Table 8).

### 3.3. Health-Related Quality of Life

Between baseline and 4 months, participants receiving the all-oral shorter regimen showed significantly greater improvement in self-assessed median health-related quality of life compared with those on the standard regimen (β 3.2, 95% CI 0.59–5.8; *p* = 0.017); however, there were no significant differences observed at the other timepoints (Table 9).

## 4. Discussion

This study provides important evidence supporting the effectiveness and safety of an all-oral shorter regimen for the treatment of rifampicin-resistant and multidrug-resistant tuberculosis (RR/MDR-TB) in Nigeria, compared to the standard of care (SOC) regimen. Although differences in treatment success between the two regimens did not reach statistical significance, the all-oral shorter regimen consistently demonstrated favourable trends, including higher proportions of treatment success and lower rates of death and loss to follow-up. These findings reinforce the potential of this regimen to improve RR/MDR-TB outcomes in programmatic settings and point to a regimen that is well tolerated by patients in Nigeria.

Our findings demonstrated that the all-oral shorter regimen achieved a higher treatment success rate at the end of treatment (81.1% vs. 76.8% for SOC), with a notably higher cure rate (80.7% vs. 68.8%). While these findings were not significant, the results are consistent with recent clinical trials and observational studies that have reported improved efficacy, tolerability, and adherence associated with shorter, fully oral regimens [20,21,22]. The advantages of this all-oral approach include shorter treatment duration, elimination of injectable toxicities, and greater convenience, all of which likely contribute to enhanced treatment adherence and patient satisfaction [23].

Despite high initial success rates, treatment outcomes declined slightly over longer follow-up due to increasing non-assessable patients, underscoring the critical need for strengthened post-treatment surveillance and support. The occurrence of treatment failures, primarily due to bacteriological reversion in the all-oral group and lack of culture conversion in the SOC group, warrants further investigation to elucidate potential contributing factors such as drug pharmacokinetics, adherence patterns, or comorbid conditions [24].

Safety profiles were broadly comparable between regimens, with no drug-related serious adverse events reported. Notably, one-third of patients experienced elevated liver enzymes and a significant proportion developed prolonged QT intervals, highlighting the importance of close clinical and laboratory monitoring to promptly identify and manage potential toxicities [25,26]. The low incidence of other adverse effects such as neuropsychiatric symptoms, anemia, and ototoxicity affirms the relative safety and tolerability of these regimens [27,28].

Mortality rates, although relatively low, remain concerning and were not attributable to treatment-related adverse events. Contributing factors such as comorbidities, treatment delays, and social determinants must be addressed to further reduce mortality in this vulnerable population [29,30,31]. The absence of prolonged hospitalizations with the all-oral regimen suggests potential benefits for decentralizing care and reducing healthcare system burdens, enhancing accessibility and patient-centred care.

While this study has generated important evidence on the effectiveness, safety, and health-related quality of life of the new shorter regimen for DR-TB in real-world, programmatic settings, it must be considered with the following limitations in mind: (1) the use of a non-randomized study design is inherently susceptible to selection bias; however, we attempted to mitigate this through cluster-level allocation and systematic enrollment of all eligible DR-TB patients; (2) we did not conduct certain exploratory analyses, such as assessing associations between disease extent, cavitation, and treatment outcomes, which were beyond the pre-specified analysis plan yet could present a more nuanced understanding of treatment effectiveness, nor did we conduct stratified analyses by age or resistance profile due to the small number of children and patients with isoniazid resistance included in the study; and (3) this study was not sufficiently powered to detect differences in rare adverse events.

In conclusion, our study substantiates the World Health Organization’s recommendation and supports the Nigerian National Tuberculosis and Leprosy Control Programme’s transition to all-oral shorter regimens for RR/MDR-TB management. The all-oral shorter regimen showed comparable effectiveness to the standard of care, with favorable trends in outcomes and a generally acceptable safety profile, supporting its continued programmatic implementation. Future efforts should prioritize robust follow-up systems, ongoing pharmacovigilance, and research into factors influencing treatment failure to optimize clinical outcomes and curb the RR/MDR-TB epidemic in Nigeria and similar settings.

## Figures and Tables

**Table 1:** Dosing schedule for the intervention arm.

Drug	Weight Group	
	30–50 kgs	>50 kg
Bedaquiline	400 mg once daily for 2 weeks, then 200 mg 3 times per week afterwards	
High-dose Levofloxacin (500 mg tablet)	750 mg daily	1000 mg daily
Clofazimine (100 mg gel capsule)	100 mg daily	
Linezolid (600 mg tablet)	600 mg daily	
Clofazimine	2–3 mg/kg daily	100 mg daily
Bedaquiline	>12 years and >33 kg: 400 mg daily for 14 days followed by 200 mg three times a week (same as adult dose) < 12 years or <33 kg: correct dose is unknown, but 6 mg/kg for 2 weeks, then 3 mg/kg afterwards may be tried	

**Table 2:** Examinations during the study period.

Investigation/Observation	Baseline	Treatment Phase (Months)									Follow-Up	
		1	2	3	4	5	6	7	8	9/12	+6	+12
Clinical evaluation												
Demographics, medical history	X											
Clinical examination	X	X	X	X	X	X	X	X	X	X	X	X
Treatment adherence		X	X	X	X	X	X	X	X	X		
Concomitant treatment		X	X	X	X	X	X	X	X	X	X	
Adverse events		X	X	X	X	X	X	X	X	X	X	
Bacteriology												
Sputum smear	X	X	X	X	X	X	X	X	X	X	X	X
Sputum culture	X	X	X	X	X	X	X	X	X	X	X	X
DST (FQ/Injectables) ^1^	X										X	X
Laboratory tests												
Hemoglobin/platelets count/white blood count ^2^	X	X	X	X	X	X	X	X	X	X		
Serum creatinine and potassium ^3^	X											
Serum liver enzymes	X	X	X	X	X	X	X	X	X	X		
Pregnancy test ^4^	X											
HIV and hepatitis B and C	Xµ											
TSH ^5^	X			X			X					
Other investigations												
Chest X-ray ^6^	X											
ECG	X	Xβ	X	X	X	X	X	X	X	X		
Visual acuity and BPNS ^7^	X	(X)	(X)	(X)	(X)	(X)	(X)	(X)	(X)	(X)		
Disability assessment	X									X	X	X

^1^ Performed for patients with any positive culture during the post-treatment follow-up or after conversion. ^2^ Performed only for patients taking linezolid. ^3^ At baseline and if clinically indicated or ECG abnormalities. ^4^ Female patients only. ^5^ Among patients receiving Pto/Eto. ^6^ Repeated as needed during follow-up for patients under suspicion of recurrence. ^7^ Continued only among patients receiving Lzd and high-dose INH/EMB; BPNS = Brief Peripheral Neuropathy Screen. Β Baseline ECG was obtained and additional ECGs were conducted at week 1 and 2 after starting treatment and thereafter monthly throughout treatment. ECG was be repeated as necessary in case of clinical suspicion of heart rhythm and conduction disturbances, or other clinical signs (e.g. dehydration and electrolyte misbalance). X just signals that the test should be performed at the certain time point. (X) Only performed for patients receiving Lzd and high-dose INH/EMB).

**Table 3:** Socio-demographic characteristics and comorbidities of participants at baseline.

	Total (N = 383) n (%)	Intervention Regimen (n = 185) n (%)	Standard of Care Regimen (n = 198) n (%)	*p*-Value ^1^
Age (years)				0.8
Median (IQR)	35 (25, 45)	35 (25, 45)	35 (26, 43)	
Age groups (years)				0.07
<15	5 (1.3)	4 (2.2)	1 (0.5)	
15–24	75 (19.6)	41 (22.2)	34 (17.2)	
25–44	204 (53.3)	87 (47.0)	117 (59.1)	
≥45	99 (25.8)	53 (28.6)	46 (23.2)	
Sex				0.4
Male	245 (64.0)	122 (65.9)	123 (62.1)	
Female	138 (36.0)	63 (34.1)	75 (37.9)	
Type of patient				0.006
New TB case	298 (77.8)	150 (81.1)	148 (74.7)	
Relapse	38 (9.9)	15 (8.1)	23 (11.6)	
Treatment after LTFU	17 (4.4)	2 (1.1)	15 (7.6)	
Treatment after failure	28 (7.3)	17 (9.2)	11 (5.6)	
Other previously treated patients	2 (0.5)	1 (0.5)	1 (0.5)	
History with TB drugs among non-new TB cases (n = 88)				0.5
Previously treated only with first-line drugs	79 (92.9)	34 (97.1)	45 (90.0)	
Previously treated with second-line drugs	3 (3.5)	1 (2.9)	2 (4.0)	
Unknown	3 (3.5)	0 (0.0)	3 (6.0)	
Missing	0	0	0	
BMI in kg/m ^2^				0.3
Median (IQR)	18.4 (16.6, 21.0)	18.6 (16.9, 21.2)	18.2 (16.2, 20.5)	
Existing neuropathy				0.09
Yes	2 (0.5)	1 (0.5)	1 (0.5)	
No	377 (98.4)	180 (97.3)	197 (99.5)	
Unknown	4 (1.0)	4 (2.2)	0 (0)	
Pregnancy test results among female patients				0.6
Not applicable	246 (64.2)	123 (66.5)	123 (62.1)	
Negative	120 (31.3)	53 (28.6)	67 (33.8)	
Positive	3 (0.8)	1 (0.5)	2 (1.0)	
Unknown/Missing	14 (3.7)	8 (4.3)	6 (3.0)	
HIV Status				0.9
Positive	38 (9.9)	18 (9.7%)	20 (10.1)	
Negative	345 (90.1)	167 (90.3%)	178 (89.9)	
Hepatitis B				<0.001
Positive	20 (5.2)	18 (9.7)	2 (1.0)	
Negative	361 (94.3)	165 (89.2)	196 (99.0)	
Unknown/Missing	2 (0.5)	2 (1.1)	0 (0.0)	
Hepatitis C				0.3
Positive	11 (2.9)	7 (3.8)	4 (2.0)	
Negative	371 (96.9)	177 (95.7)	194 (98.0)	
Unknown/Missing	1 (0.3)	1 (0.5)	0 (0.0)	
Diabetes				0.2
Yes	10 (2.6)	7 (3.8)	3 (1.5)	
No	361 (94.3)	166 (89.7)	195 (98.5)	
Missing	12(3.1)	12 (6.5)	0 (0.0)	
Hemoglobin (g/dL)				0.4
Mean (SD)	11.06 (2.19)	11.14 (2.12)	10.99 (2.26)	
Median (IQR)	11.00 (9.60, 12.50)	11.00 (9.90, 12.60)	11.00 (9.50, 12.30)	
Glucose (mg/dL)				<0.001
Mean (SD)	96 (46)	93 (52)	99 (40)	
Median (IQR)	91 (78, 100)	84 (73, 96)	94 (84, 104)	
ALT/SGPT (U/L)				0.4
Mean (SD)	19 (22)	18 (13)	20 (28)	
Median (IQR)	14 (8, 22)	15 (9, 21)	13 (8, 25)	
Creatinine (mg/dL)				<0.001
Mean (SD)	0.81 (0.58)	0.73 (0.45)	0.90 (0.68)	
Median (IQR)	0.70 (0.60, 0.90)	0.63 (0.58, 0.80)	0.80 (0.60, 1.00)	
Disability assessment (mMRC)				0.5
*“I only get breathless with a strenuous exercise”*	247 (64.8)	122 (66.3)	125 (63.5)	
*“I get short of breath when hurrying on the level or walking up a slight hill”*	91 (23.9)	41 (22.3)	50 (25.4)	
* “I walk slower than people of the same age on the level because of breathlessness or have to stop for breath when walking at my own pace on the level”*	26 (6.8)	12 (6.5)	14 (7.1)	
* “I stop for breath after walking about 100 yards or after a few minutes on the level”*	12 (3.1)	8 (4.3)	4 (2.0)	
* “I stop for breath after walking about 100 yards or after a few minutes on the level”*	5 (1.3)	1 (0.5)	4 (2.0)	
Missing	2	1	1	

^1^ Wilcoxon rank sum test; Fisher’s exact test; Pearson’s Chi-squared test.

**Table 4:** TB disease characteristics of patients.

	Total (N = 383) n (%)	Intervention Regimen (n = 185) n (%)	Standard of Care Regimen (n = 198) n (%)	*p*-Value ^1^
Sputum smear				0.01
Negative	185 (48.6)	84 (45.7)	101 (51.3)	
Scanty	23 (6.0)	8 (4.3)	15 (7.6)	
1+	87 (22.8)	50 (27.2)	37 (18.8)	
2+	40 (10.5)	13 (7.1)	27 (13.7)	
3+	46 (12.1)	29 (15.8)	17 (8.6)	
Missing	2	1	1	
Culture results (n = 391)				0.3
Positive	223 (59.5)	114 (62.0)	109 (57.1)	
Negative	152 (40.5)	70 (38.0)	82 (42.9)	
Missing	8	1	7	
Rifampicin sensitivity				0.9
Resistant	383 (100.0)	185 (100.0)	198 (100.0)	
Sensitive	0 (0.0)	0 (0.0)	0 (0.0)	
Fluoroquinolone sensitivity				0.05
Sensitive	325 (84.9)	164 (88.6)	161 (81.3)	
Resistant	0 (0.0)	0 (0.0)	0 (0.0)	
Unknown/missing	58 (15.1)	21 (11.4)	37 (18.7)	
Isoniazid sensitivity				0.002
Resistant	8 (2.1)	7 (3.8)	1 (0.5)	
Sensitive	184 (48.0)	75 (40.5)	109 (55.1)	
Unknown/missing	191 (49.9)	103 (55.7)	88 (44.4)	
Cavitation on CXR				<0.001
Yes	175 (45.7)	129 (69.7)	46 (23.2)	
No	180 (47.0)	48 (25.9)	132 (66.7)	
Unknown/missing	28 (7.3)	8 (4.3)	20 (10.1)	
Extent of the disease				0.001
A(<25%)	110 (32.2)	50 (28.6)	60 (35.9)	
B(25–49%)	142 (41.5)	83 (47.4)	59 (35.3)	
C(>50%)	37 (10.8)	25 (14.3)	12 (7.2)	
Unknown	53 (15.5)	17 (9.7)	36 (21.6)	

^1^ Wilcoxon rank sum test; Fisher’s exact test; Pearson’s Chi-squared test.

**Table 5:** Treatment outcomes at the end of the six-month follow-up.

	Total (N = 383) n (%)	Intervention Regimen (N = 183) n (%)	Standard of Care Regimen (N = 198) n (%)	Adjusted RR [95% CI]; *p* Value ^a^
Favourable	287 (74.9%)	149 (80.5%)	138 (69.7%)	1.13 [0.85,1.49]; 0.4
Cured	277 (72.3%)	144 (77.8%)	133 (67.2%)	
Treatment completed	10 (2.6%)	5 (2.7%)	5 (2.5%)	
Unfavourable	78 (20.4%)	34 (18.4%)	44 (22.2%)	
Died during treatment	29 (7.6%)	12 (6.5%)	17 (8.6%)	
Treatment failure	8 (2.1%)	5 (2.7%)	3 (1.5%)	
Loss of follow-up	39 (10.2%)	16 (8.6%)	23 (11.6%)	
Recurrent episode	2 (0.5%)	1 (0.5%)	1 (0.5%)	
Not assessable	18 (4.7%)	2 (1.1%)	16 (8.1%)	
Cured without permanent disability ^b^	229 (70.7%)	98 (73.7%)	131 (68.6%)	

^a^ The standard treatment is the reference; adjusted by age group, type of patient, hepatitis B status, platelet results, glucose, creatinine, presence of cavities at chest x-ray, isoniazid sensitivity, and sputum smear results. ^b^ Patients for whom six-month follow-up status is unknown.

**Table 6:** Treatment outcomes at the end of the treatment phase.

End of Treatment Outcome	Total (n = 383) n (%)	Intervention Regimen (n = 185) n (%)	Standard of Care Regimen (n = 198) n (%)
Favourable outcome	302 (78.9)	150 (81.1)	152 (76.8)
Cured	284 (74.2)	145 (78.4)	139 (70.2)
Treatment completed	18 (4.7)	5 (2.7)	13 (6.6)
Died during treatment	29 (7.6)	12 (6.5)	17 (8.6)
Lost to follow-up	39 (10.2)	16 (8.6)	23 (11.6)
Treatment failure	8 (2.1)	5 (2.7)	3 (1.5)
Not evaluated	5 (1.3)	2 (1.1)	3 (1.5)
Treatment adherence	374 (97.7)	178 (92.7)	196 (94.2)

**Table 7:** Number of episodes of AEs and number of different types of AEs/patients.

	Total	Intervention Regimen (N = 185) n (%)	Standard of Care Regimen (N = 198) n (%)
Total number of AEs reported during the treatment phase	1277	638	639
Total number of individual patients reporting at least one AE of interest	344	161 (87.0)	183 (92.4)
Number of episodes of AEs of interest per patient			
Median (IQR)	4 (2, 5)	3 (2, 4)	3 (2, 5)
Minimum–maximum	1–12	1–11	1–12
Number of different types of AEs of interest per patient			
Median (IQR)	2 (2, 3)	2 (2, 3)	2 (2, 3)
Minimum–maximum	1–5	1–5	1–5

**Table 8:** Serious adverse events (SAEs).

	Total	Intervention Regimen (N = 183) n (%)	Standard of Care Regimen (N = 198) n (%)	Adjusted ^a^ RR ^b^ [95% CI]; *p* Value
Patients who had at least one serious adverse event ^b^	32 (34.4%)	12 (6.6%)	20 (10.0)	0.48 [0.21, 1.14]; 0.10
Had at least one of the following serious adverse events				
Death	28 (82.3%)	11 (91.7%)	17 (81.0%)	
Life-threatening experience	1 (2.9%)	1 (8.3%)	0 (0.0%)	
Any hospitalization or prolongation of hospitalization	4 (11.8%)	0 (0.0%)	4 (19.0%)	
Persistently or significantly disabling event	1 (2.9%)	1 (7.7%)	0 (0.0%)	
SAE related to the use of medication				
Related	0 (0%)	0 (0%)	0 (0%)	
Not related	23 (69.7%)	11 (91.7%)	12 (57.1%)	
Insufficient data to assess	10 (30.3%)	1 (8.3%)	9 (42.9%)	

^a^: The standard treatment is the reference; adjusted by age group, type of patient, hepatitis B status, platelet results, glucose, creatinine, presence of cavities at chest X-ray, isoniazid sensitivity, and sputum smear results. ^b^: Total number of SAEs = 34 in 32 patients (2 patients experienced SAE twice), and there were, in total, 4 episodes of hospitalization or prolongation of hospitalization, but 1 patient experienced it twice.

**Table 9:** HQoL measurements over time using EQ-VAS.

	Intervention Regimen (n = 185)	Standard of Care Regimen (n = 198)	β [95% CI]; *p*-Value
Between baseline and 4 months			3.2 [0.59, 5.8]; 0.017
Median (IQR)	10.0 (5.0, 15.0)	5.0 (5.0, 10.0)	
Missing(%)	41.0 (22.2%)	45.0 (22.7%)	
Between baseline and end of treatment			−0.24 [3.5, 3.0]; 0.9
Median (IQR)	15.0 (10.0, 15.0)	10.0 (9.0, 20.0)	
Missing(%)	56.0 (30.3%)	60.0 (30.3%)	
Between baseline and end of follow-up			1.7 [−2.2, 5.5]; 0.4
Median (IQR)	15.0 (10.0, 20.0)	10.0 (9.0, 20.0)	
Missing(%)	82.0 (44.3%)	76.0 (38.4%)	
Between end of treatment and end of follow-up			
Median (IQR)	0.0 (0.0, 0.8)	0.0 (−2.0, 2.0)	
Missing (%)	83.0 (44.9%)	77.0 (38.9%)	

## Data Availability

The datasets presented in this article are not readily available because specific consent from participants was not granted for the sharing of individual data. Requests to access the datasets should be directed to the corresponding author, mentioning the objectives and intended data analysis plan. We note that the generic protocol and associated data collection tools used to guide this study is publicly available from the following site: https://tdr.who.int/activities/shorrt-research-package (accessed on 5 December 2025).

## Abbreviations

The following abbreviations are used in this manuscript:

ADR	Adverse Drug Reaction
AE	Adverse Event
AF	Acid-Fast
AFB	Acid-Fast Bacilli
ALAT/SGPT	Alanine Aminotransferase/Serum Glutamic Pyruvic Transaminase
APC	Article Processing Charge
BMI	Body Mass Index
BPNS	Brief Peripheral Neuropathy Screen
BPaL	Bedaquiline, Pretomanid, and Linezolid (all-oral regimen)
Bdq	Bedaquiline
CI	Confidence Interval
COPD	Chronic Obstructive Pulmonary Disease
CRF	Case Report Form
CXR	Chest X-Ray
Cfz	Clofazimine
DOT	Directly Observed Treatment
DR-TB	Drug-Resistant Tuberculosis
DST	Drug Susceptibility Testing
ECG	Electrocardiogram
EMB	Ethambutol
EQ-5D	EuroQol 5-Dimension Health Questionnaire
EQ-VAS	EuroQol Visual Analogue Scale
Eto	Ethionamide
FQ	Fluoroquinolone
HIV	Human Immunodeficiency Virus
Hh	High-dose Isoniazid
INH	Isoniazid
IQR	Interquartile Range
LPA	Line Probe Assay
LTFU	Lost to Follow-Up
Lzd	Linezolid
MDR-TB	Multidrug-Resistant Tuberculosis
Mfx	Moxifloxacin
NHREC	National Health Research Ethics Committee
NTBLCP	National Tuberculosis and Leprosy Control Programme
Pre-XDR-TB	Pre-Extensively Drug-Resistant Tuberculosis
QtcF/QTc	Corrected QT Interval (Fridericia formula)
REDCap	Research Electronic Data Capture (database software)
RR-TB	Rifampicin-Resistant Tuberculosis
SAE	Serious Adverse Event
SD	Standard Deviation
SOC	Standard of Care
TB	Tuberculosis
TDR	Special Programme for Research and Training in Tropical Diseases
TSH	Thyroid-Stimulating Hormone
WHO	World Health Organization
XDR-TB	Extensively Drug-Resistant Tuberculosis
Xpert MTB/RIF	GeneXpert Mycobacterium tuberculosis/Rifampicin Resistance Assay
hLfx	High-dose Levofloxacin
mMRC	Modified Medical Research Council (Dyspnea Scale)
msec	Milliseconds

## Appendix A

**Table A1:** Frequency of AEs of interest and grading.

		Treatment Regimen	
	Overall, N = 1277 ^1^	All-Oral Shorter N = 638 ^1^	Standard N = 639 ^1^
Type of AE of interest			
Anemia	269 (21.1%)	145 (22.7%)	124 (19.4%)
Thrombopenia	145 (11.4%)	70 (11.0%)	75 (11.7%)
Leukopenia	17 (1.3%)	13 (2.0%)	4 (0.6%)
Elevated liver enzymes	481 (37.7%)	229 (35.9%)	252 (39.4%)
Prolonged QtcF	353 (27.6%)	172 (27.0%)	181 (28.3%)
Peripheral neuropathy	7 (0.5%)	7 (1.1%)	0 (0.0%)
Anxiety	2 (0.2%)	0 (0.0%)	2 (0.3%)
Depression	1 (0.1%)	1 (0.2%)	0 (0.0%)
Optic neuritis	1 (0.1%)	1 (0.2%)	0 (0.0%)
Ototoxicity	1 (0.1%)	0 (0.0%)	1 (0.2%)
Grade of AE of interest			
Grade 1	586 (45.9%)	301 (47.2%)	285 (44.6%)
Grade 2	488 (38.2%)	245 (38.4%)	243 (38.0%)
Grade 3	158 (12.4%)	72 (11.3%)	86 (13.5%)
Grade 4	45 (3.5%)	20 (3.1%)	25 (3.9%)

^1^ n (%).

**Table A2:** Proportion of patients experiencing each AE of interest.

		Treatment Regimen		
	Overall, N = 380 ^1^	All-Oral Shorter N = 181 ^1^	Standard N = 199 ^1^	*p*-Value ^2^
Anemia	184 (48.4%)	93 (51.4%)	91 (45.7%)	0.4
Thrombocytopenia	111 (29.2%)	47 (26.0%)	64 (32.2%)	0.4
Leukopenia	14 (3.7%)	10 (5.5%)	4 (2.0%)	0.2
Elevated liver enzymes	279 (73.4%)	121 (66.9%)	158 (79.4%)	0.008
QTcF prolongation	211 (55.5%)	101 (55.8%)	110 (55.3%)	>0.9
Peripheral neuropathy	7 (1.8%)	7 (3.9%)	0 (0.0%)	0.005
Anxiety	1 (0.3%)	0 (0.0%)	1 (0.5%)	>0.9
Depression	1 (0.3%)	1 (0.6%)	0 (0.0%)	0.5
Optic neuritis	1 (0.3%)	1 (0.6%)	0 (0.0%)	0.5
Ototoxicity	1 (0.3%)	0 (0.0%)	1 (0.5%)	>0.9

^1^ n (%), ^2^ Fisher’s exact test; Pearson’s Chi-squared test.
